# Towards a shared diagnostic approach for pediatric short stature: a Delphi consensus of the Italian Society of Pediatrics and the Italian Society for Pediatric Endocrinology and Diabetology

**DOI:** 10.1186/s13052-025-02190-6

**Published:** 2026-01-10

**Authors:** Chiara Mameli, Stefano Cianfarani, Luisa De Sanctis, Mohamad Maghnie, Malgorzata Wasniewska, Francesco Chiarelli, Maria Felicia Faienza, Maria Elisabeth Street, Giampaolo De Luca, Cinzia Sacchetti, Massimiliano Orso, Barbara Polistena, Rino Agostiniani, Valentino Cherubini, Annamaria Staiano, Mariacarolina Salerno

**Affiliations:** 1 Department of Pediatrics, Vittore Buzzi Children’s Hospital, Università degli Studi di Milano, Milan, Italy; 2 Department of Biomedical and Clinical Science, Università di Milano, Milan, Italy; 3 Department of Systems Medicine, University of Rome ‘Tor Vergata’, Rome, Italy; 4 Endocrinology & Diabetes Unit, ‘Bambino Gesù’ Children’s Hospital, Rome, Italy; 5 Department of Women’s and Children’s Health, Karolinska Institutet, Stockholm, Sweden; 6 Department of Public Health and Pediatric Sciences, Regina Margherita Childrens’ Hospital, University of Torino, Torino, Italy; 7 Department of Pediatrics, IRCCS Istituto Giannina Gaslini, Genova, Italy; 8 Department of Neuroscience, Rehabilitation, Ophthalmology, Genetics, Maternal and Child Health - University of Genova, Genova, Italy; 9 Department of Human Pathology in Adulthood and Childhood, University of Messina, Messina, Italy; 10 Department of Pediatrics, University of Chieti, Chieti, Italy; 11 Pediatric Unit, Department of Precision and Regenerative Medicine and Ionian Area, University of Bari “Aldo Moro”, Bari, Italy; 12 Department of Medicine and Surgery, University of Parma, Parma, Italy; 13 Unit of Paediatrics, University Hospital of Parma, Parma, Italy; 14 Azienda Sanitaria Provinciale (ASP) Cosenza, Cosenza, Italy; 15 A.Fa.D.O.C. APS, Vicenza, Italy; 16 C.R.E.A. Sanità (Centre for Applied Economic Research in Healthcare), Rome, Italy; 17 Department of Pediatrics, San Jacopo Hospital, Pistoia, Italy; 18 Department of Women’s and Children’s Health, G. Salesi Hospital, Ancona, Italy; 19 Pediatric Section- Department of Translational Medical Sciences, University Federico II, Naples, Italy

**Keywords:** Short stature, Children, Growth, Consensus

## Abstract

**Background:**

Short stature is a common reason for referral in pediatric practice, but significant variability exists in diagnostic approaches and referral criteria. Therefore, the aim of this consensus was to improve the management of children with short stature through the knowledge and expertise of various Italian centers through a Delphi-consensus process.

**Methods:**

A multidisciplinary scientific board of 11 experts and 1 patient advocate were involved in a consensus process and used the Delphi method. The scientific board identified 4 key domains regarding short stature: clinical evaluation, biochemical assessment, imaging and genetic investigation. Two literature searches were conducted. The first search aimed to identify international guidelines and consensus on the diagnosis of short stature in children. The second search sought to retrieve clinical studies focusing on the four domains. The results of the literature searches were evaluated by the scientific board and a total of 14 statements were drafted and submitted to a panel of 39 pediatricians and pediatric endocrinologists using a two-round Delphi method conducted between February and March 2025. Consensus was defined as ≥ 80% agreement or disagreement (scores 7–9 or scores 1–3 on a 9-point Likert scale).

**Results:**

In the first Delphi round, 34 out of 39 experts responded (response rate 87%), and consensus was reached on 11 out of 14 statements. The remaining 3 statements were revised by the scientific board and re-submitted. In the second round, 34 of 34 eligible experts responded (100%), and consensus was achieved for one additional statement. Overall, a strong agreement was reached for 12 out of 14 statements.

**Conclusion:**

This consensus provides valuable insights and recommendations to guide pediatricians in approaching a child with short stature. By addressing clinical, biochemical, imaging and genetic domains this consensus provides a model of growth monitoring that may result in early detection and appropriate management of children with short stature.

**Supplementary Information:**

The online version contains supplementary material available at 10.1186/s13052-025-02190-6.

## Introduction

Short stature is a common reason for referral in pediatric practice [1]. Diagnosing short stature in children involves more than just measuring height. A systematic approach is essential to allow a correct diagnosis, avoid diagnostic errors and ensure appropriate treatment. Careful clinical assessment and accurate interpretation of growth, biochemical, imaging, and genetic data, are essential. Avoiding inaccurate diagnoses and unnecessary treatments is crucial and should be guided by up-to-date, evidence-based guidelines.

Scientific societies and international groups have produced different clinical standards for the initial assessment, investigation, and testing of children with short stature. Most of these standards have been achieved with consensus based on expert opinions [2–7]. Only one guideline was published using the PICO model [7]. Given the lack of uniformity in diagnostic and management criteria, this consensus has been proposed to improve the care of children by harnessing the knowledge and expertise of Italian pediatricians and endocrinologists through a Delphi-like consensus process.

This consensus statement provides insights and recommendations to guide pediatricians in approaching a child with short stature. By addressing clinical, biochemical, imaging, and genetic domains, the consensus aims to give a model of growth monitoring and improving management of short stature in children.

## Methods

A multidisciplinary scientific board of experts in short stature was established. The panel consisted of 10 pediatric endocrinologists across all Italian Regions, one family pediatrician, one representative from the Italian patient association A.Fa.D.O.C. onlus and two experts in Delphi methodology.

Two separate literature searches were conducted to support the scientific board in the development of the consensus statements. The first literature search aimed to identify international guidelines and consensus documents on the diagnosis of short stature in children. The second literature search focused on identifying clinical studies describing the diagnostic process.

The results of both literature searches were presented to the scientific board and discussed during plenary meetings. The topics relevant for the analysis were defined and the related statements were prepared through a series of online meetings.

A panel of 39 Italian pediatricians working in pediatric endocrinology centers, in pediatric hospitals or as family pediatrician from all the Italian Regions were involved in a consensus process, using the Delphi method, to reach an agreement on the list of statements on the management of patients with short stature.

### Literature search

The first systematic literature search was conducted on March 30, 2024, using PubMed, Embase, Web of Science, and Google Scholar data bases to identify relevant guidelines and consensus documents on the diagnosis of short stature in children. The full search strategy is reported in Supplementary File 1. The study selection process was carried out independently by two reviewers in two phases. Initially, titles and abstracts were screened based on predefined inclusion criteria: English-language guidelines and consensus statements focusing on the diagnosis of short stature in pediatric populations. Subsequently, potentially eligible full-text articles were assessed. Any disagreements between reviewers were resolved through discussion. Data extraction was performed by one reviewer and verified by a second. The literature selection process is illustrated in Supplementary File 2 (PRISMA 2020 Flow Diagram). A list of excluded studies with reasons for exclusion is provided in the Supplementary File 3.

The second literature review included four separate searches, one for each diagnostic domain (clinical, biochemical, imaging, genetic), conducted on July 1, 2024. As with the first review, study selection was performed in duplicate by couples of review authors. Disagreements were resolved by consensus or, if needed, by a third reviewer. Data extraction was conducted by one reviewer and verified by another. The results of these systematic reviews will be reported in separate scientific publications.

The literature search aimed at identifying guidelines and consensus documents yielded 202 records. After removing duplicates, 141 records were screened by title and abstract, and 18 full-text articles were assessed for eligibility. Of these, 12 were excluded, and 6 were included in the final analysis [2–7]. Among the included studies, 3 were consensus statements [2–4], while the remaining 3 were described as guidelines [5–7]. However, these guidelines were mostly based on expert opinion, rather than on a systematic literature review and a robust methodology framework, such as the GRADE approach for assessing the quality of evidence. The paper by Wit et al. [7] was the only document that described an evidence-based approach and explicitly reported the PICO questions that were investigated. The characteristics of included studies are described in Table 1.

**Table 1:** Characteristics of consensus statement documents and guidelines on pediatric short stature

Study ID	Design	Country	Population	Clinical area / Topic
Cohen 2008 [2]	Consensus statement	International	Children with idiopathic short stature	Diagnosis and treatment
Collett-Solberg 2019 [3]	Consensus statement	International (14 countries)	• Diagnosis: children with short stature and/or growth deceleration. • Treatment: children with growth hormone deficiency.	Diagnosis, genetics, and therapy
Corripio-Collado 2022 [4]	Consensus statement	Spain	• Children with short stature; • Small-for-gestational-age (SGA) children.	1) Diagnosis; 2) Monitoring of the small for gestational age patient; 3) Growth hormone treatment; 4) Treatment adherence.
Seaver 2009 [5]	Practice guideline	United States	• Children with short stature; • Fetuses with intrauterine growth restriction	Genetic evaluation
van Dommelen 2020 [6]	Guideline	The Netherlands	Children and adolescents with short stature (or growth faltering) or tall stature (or accelerated growth)	Early referral in preventive child healthcare
Wit 2019 [7]	Guideline	The Netherlands	Children with growth failure (short stature and/or growth faltering)	Stepwise diagnostic approach

A summary of the included consensus statements and guidelines, together with the results of the literature review across the four domains (clinical, biochemical, imaging, and genetic), was presented to the scientific board. This material served as the basis for identifying areas of uncertainty and variability in clinical practice, and for the subsequent formulation of the consensus statements.

### Areas of debate

After extensive revision of the literature and based on clinical experience of the scientific board, areas of uncertainty and variability in the management of the child with short stature were identified and translated into statements.

Four main topics were identified: (1) clinical evaluation; (2) biochemical assessment (3) imaging; (4) genetic investigation and working subgroups were formed for each topic.

A total of 14 statements were formulated for the purpose of the Delphi process and discussed by the scientific board through a series of online meetings (Table 2). Statements were preceded by a brief introduction to frame the context and rationale.

### Delphi study design

A two-round Delphi method was employed to reach consensus among a panel of experts [8]. This method has previously been used by the authors in similar studies [9, 10]. The Delphi method is a structured technique aimed at leading a group of experts to reach consensus on complex or uncertain topics through a series of questionnaires, interspersed with controlled feedback. The process guarantees anonymity of individual responses, minimizing potential biases due to dominance or group conformity (also known as groupthink) [8]. In addition, the method allows respondents to modify their initial judgements based on aggregated feedback from the previous round. Finally, respondents can provide qualitative comments on statements that do not achieve strong convergence, offering useful insights for the analysis of disagreements [11].

The Delphi panel received an e-mail invitation to participate in the study and completed the two survey rounds online. Responses were analysed using descriptive statistics.

**Table 2:** Statements proposed to the panel of clinical experts during the first round of the Delphi process

S1	**TOPIC 1 - CLINICAL EVALUATION** Early recognition of growth abnormalities is crucial for identifying stunted growth and potential abnormal short stature in pediatric patients. The following criteria are defined for early detection: • a height less than − 2 standard deviation score (SDS) (or below the 3rd percentile) compared to the mean height for a given age, sex, and population group AND/OR • inadequate short stature relative to the corresponding genetic target height AND/OR • decreased height velocity (< -1 SDS) over the last 6 months
S2	The Harpenden stadiometer is the most accurate instrument for measuring stature. Alternatively, a simple wall-mounted stature meter, such as a millimeter tape attached to the wall with a sliding plastic rod placed overhead, can be used. For children under the age of two, length should be measured in the supine position using an infantometer. All instruments should be regularly checked for accuracy and precision. **Length/height measurements must be reported using the appropriate charts for the reference population and gender: INeS charts for newborns**,** WHO growth charts for infants (< 2 years)**,** and Cacciari or CDC growth charts for children aged 2 years or older. The CDC charts**,** derived from a multiethnic population**,** are recommended for non-Caucasian subjects. To calculate height SDS and growth velocity**,** we suggest using the ISPED growth calculator 4 tool (available for download at** www.siedp.it**) or other electronic child growth calculators.**
S3	Growth velocity (GV) is essential for assessing whether longitudinal growth is proceeding regularly and adequately. It is calculated using measurements taken 6–12 months apart. GV is highest during the 1st year of life (22–24 cm), decreases in the 2nd year (10 cm/year), and then stabilizes at around 5–6 cm/year in the years prior to puberty. It typically undergoes a physiological slowdown in the year before the pubertal growth spurt, which is more pronounced in individuals with delayed puberty. During the pubertal growth spurt, GV peaks at an average to 10–12 cm/year. GV should be calculated and plotted on Tanner charts, or the SDS should be calculated using an electronic tool. **Growth velocity is a sensitive indicator of growth impairment**,** and should be regularly evaluated in all children**,** according to the age at least annually. If growth velocity is below − 1 SDS**,** it should be re-assessed after 6 months.**
S4	The identification of newborns with impaired fetal growth is crucial, as they are at high risk of post-natal growth failure. Early identification allows for growth risk prediction, height prediction assessment, and timely intervention. Neonatal charts specific to Italian singletons (first- and second-born) and twins born between 23 gestational weeks, known as INeS charts, are available for this purpose. **In children born small for gestational age (SGA)**,** as established on the InesCharts**,** a standard growth evaluation should be performed every 6 months during the first 2 years of life**,** then annually. By the 4th year of life**,** children born SGA should be referred for a pediatric endocrine evaluation if their height is below − 2.5 SDS and their height velocity is less than 0 SDS.** **TOPIC 2 - BIOCHEMICAL ASSESSMENT**
S5	In a child where medical history and clinical examination do not clearly indicate organ system disorders or secondary growth failure, investigations should be conducted to assess glucose metabolism, anemia, infection/inflammation, kidney-liver and thyroid function, calcium-phosphate metabolism, screening for celiac disease, and the GH-IGF axis. **The following blood tests should be considered mandatory in primary care: full blood count**,** fasting blood glucose**,** creatinine**,** electrolytes (sodium**,** potassium)**,** liver function tests (transaminase**,** serum gamma-glutamyl transferase)**,** C reactive protein**,** calcium**,** phosphorus**,** alkaline phosphatase**,** screening for celiac disease**,** venous blood gas analysis (below the age of 2 years)**,** IGF1**,** TSH**,** FT4**,** FSH in females under 2 years**,** or over 9 years of age**,** and urine analysis**.
S6	Laboratory investigations should help to identify the most appropriate setting for evaluating growth failure, guiding whether further assessment should be in primary care, or at a pediatric endocrinology center, or if a different specialized facility is needed. **Whenever growth failure is present and/or abnormal changes in IGF1**,** TSH**,** FT4**,** FSH**,** calcium**,** phosphorus**,** and alkaline phosphatase are observed**,** the child should be referred to a pediatric endocrinology specialist**,** together with the individual’s growth curve and bone age.** **TOPIC 3 - IMAGING**
S7	Bone age (BA) assessment is a crucial component in the evaluation of a child with short stature. Hand and wrist X-rays are key indicators of biological age and can be influenced by factors such as gender, nutrition, metabolic, genetics, social factors, acute or chronic diseases, and endocrine dysfunction. **A left-hand and wrist radiograph to assess bone age is essential for accurately evaluating a child with short stature and/or impaired growth. It should be ordered by the Pediatrician before referring the child with suspected short stature or growth disturbances to a pediatric endocrinologist.**
S8	Skeletal dysplasias (SDs) are a diverse and heterogeneous group of genetic conditions characterized by abnormalities in the growth, development, differentiation, and maintenance of cartilage and bone structures. This group includes 771 genetic conditions, classified into 41 groups, involving 552 distinct genes. The incidence of SDs is approximately 1 in every 3,000 to 1 in every 5,000 live births, representing around 5% of newborns with congenital malformations. However, many of these dysplasias are often underdiagnosed, primarily due to a lack of suspicion in milder forms, that do not show obvious skeletal alterations. Often, the pediatric endocrinologist is one of the first healthcare professionals to evaluate a child with suspected SD, especially due to proportionate or disproportionate short stature. Identifying key clinical features (disproportionality), conducting appropriate initial screening tests, and ensuring referral for multidisciplinary follow-up are all essential for accelerating diagnosis and providing family support. **A skeletal survey should be considered as a second-line evaluation in individuals with a phenotype suggestive of skeletal dysplasia**,** such as those with disproportionate short stature.**
S9	Magnetic resonance imaging (MRI) without contrast, using T2- DRIVE sequences of the hypothalamic–pituitary region and the entire brain (forebrain, midbrain and hindbrain) is strongly recommended for children and adolescents with severe short stature and GH testing that supports a diagnosis of GH deficiency (GHD), in children and adolescents with multiple pituitary hormone deficiency. In children with isolated GHD and severe short stature this could be due to anatomical defects of the hypothalamic-pituitary region, brain tumors or other central nervous system disorders that must be detect. This imaging plays a key role in assessing the likelihood of other pituitary deficiencies, determining the potential need for genetic testing, and predicting the likelihood of persistent GHD. **In neonates**,** infants**,** children**,** and adolescents diagnosed with hypopituitarism**,** an MRI without contrast using T2- DRIVE sequences of the hypothalamic–pituitary region and the entire brain**,** is highly recommended.**
S10	Skeletal dysplasias can often be detected during obstetric ultrasound. Approximately 80–90% of cases of achondroplasia are suspected and diagnosed before birth, typically around a median gestational age of 29–32 weeks. Additionally, antenatal ultrasound can identify brain malformations such as agenesis of the septum pellucidum (ASP), a potential indicator of the rare midline anomaly known as septo-optic dysplasia (SOD), which occurs in about 1 in 10,000 live births. SOD is characterized by a range of features including ASP, hypoplasia of one or both optic nerves, pituitary anomalies and endocrine dysfunction. **The Pediatrician should be aware that bone dysplasia can be suspected in utero through routine ultrasound. Additionally**,** antenatal ultrasound may also raise suspicion of septo-optic dysplasia. Early identification of these conditions can lead to improved management and outcomes for affected infants.** **TOPIC 4 - GENETIC INVESTIGATION**
S11	Pediatric endocrinologists are skilled in managing children with short stature of both genetic and non-genetic origin, offering the best care through a personalized diagnostic and therapeutic approach. **Any pediatrician should refer a child with short stature and suspected genetic causes to a Pediatric Endocrinology center**,** rather than to laboratories or clinical geneticists.**
S12	Short stature may be the only clinical feature in girls with Turner syndrome, a condition characterized by the presence of only one X chromosome and the complete or partial absence of the second sex chromosome. Unexplained short stature, left-sided outflow congenital heart defects, unexplained delay in puberty, primary and secondary amenorrhea, failure to progress puberty and characteristic facial and physical features are all indications for genetic testing. Other minor and less specific features may be present including sensorineural hearing loss, renal abnormalities, Madelung deformity, neurocognitive problems, and additional congenital heart defects. **Any pediatrician may order a karyotype (with a minimum of 30 metaphases counted) in girls with unexplained short stature**,** especially if associated with other characteristic signs of Turner syndrome before referring her to a secondary or tertiary care center.**
S13	Recent advances in genetics have provided new insights into the etiology of various conditions of short stature, leading to a final diagnosis and helping avoid unnecessary laboratory and endocrine tests, as well as ineffective and costly treatments. A combined approach of systematic phenotyping (also looking for dysmorphic clinical signs, body disproportions and possible cognitive defects), targeted genetic testing, and whole-exome sequencing (WES, with trio whenever possible) enables the identification of the underlying cause of short stature in more than 30% of cases. This approach allows physicians to improve diagnosis, treatment, and genetic counseling. **Genetic testing should be considered for any child with short stature when a detailed history**,** physical examination**,** laboratory results**,** and radiological findings point out to a possible genetic cause.**
S14	Due to the progressive reduction in costs and the increasing availability of easily accessible laboratory facilities, genetic testing is becoming central to the evaluation of a child with short stature. WES significantly improves the diagnostic yield and, as a result, enhances care for patients with short stature. A wide range of genetic variants have been linked to hypopituitarism. The most common pathogenic genetic variants causing non-growth hormone (GH)-dependent short stature include the ACAN, BMP2, FBN1, FGFR3, IHH, IGF-I receptor, NPPC, NPR2, PAPPA2, PTPN11, and SHOX genes. **Every pediatrician should recognize the importance of genetic testing when assessing a child with short stature and stay informed on the most common genetic tests available.**

ASP: agenesis of the septum pellucidum; BA: Bone age; CDC: Centers for Disease Control and Prevention; GHD: growth hormone deficiency; GV: Growth velocity; ISPED: Italian Society for Pediatric Endocrinology and Diabetology; MRI: Magnetic resonance imaging; S:statement; SGA: small for gestational age; SDS: standard deviation score; SDs: Skeletal dysplasias; SOD: septo-optic dysplasia; WES: whole-exome sequencing; WHO: World Health Organization

### Questionnaire and survey

The Delphi process was launched on February 19, 2025, and completed on February 26, 2025. Panelists used a dedicated online platform to participate and a timeline of 7 calendar days was set to provide answers for each round. A further 7 days were granted after a reminder e-mail. Two reminders were sent to gather as many responses as possible. The agreement was defined using a 9-point Likert scale, where scores from 1 to 3 indicated little or no agreement, scores from 4 to 6 indicated moderate agreement, scores from 7 to 9 indicated full agreement with each proposed statement. The cut-off for consensus was set at a minimum of 80% of the number of respondents, meaning that strong disagreement or agreement was considered reached if at least 80% of participants had assigned scores in the range 1–3 or 7–9 to that statement, respectively [8]. Statements with average scores within the intermediate range, meaning 4–6 (corresponding to “moderate agreement”), were not considered indicative of consensus. According to the protocol, the statements for which the level of agreement/disagreement did not reach the threshold were submitted for a second round, in which only the respondents to the first round were invited to participate. Results of the first round were shared with the respondents before the second round.

## Results

**Fig. 1:**
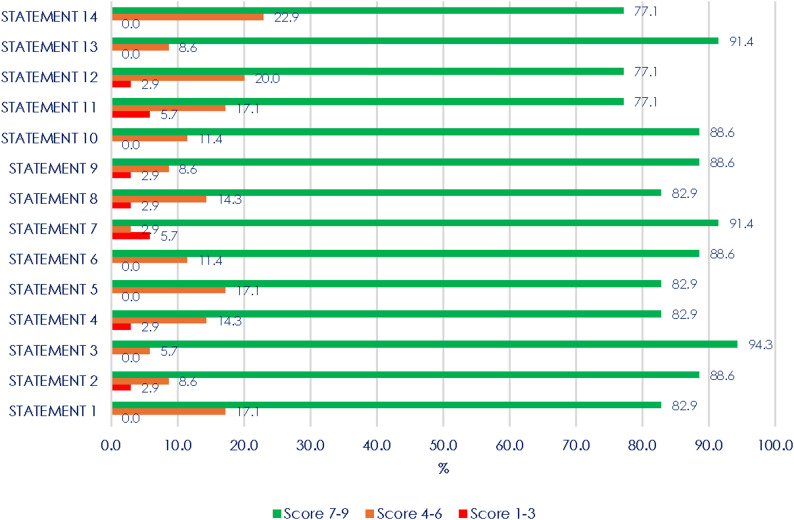
Scores of the proposed statements (first Delphi round). Results are presented in %

In the first round of the Delphi process, 34 out of 39 clinical experts invited to participate responded (response rate 87%). Among the respondents, the majority were pediatric endocrinologists (Appendix 1). The 11 statements (Ss) for which strong agreement was reached were S1, S2, S3, S4, S5, S6, S7, S8, S9, S10, and S13. It is worthwhile to highlight that none of the statements had a score within the disagreement range. (Fig. 1)

The three statements that did not reach the predefined threshold for consensus - S11, S12, and S14 - were submitted for the second round of the survey.

On the basis of the respondents’ suggestions, the 3 statements have been modified from the scientific board as in Table 3.

The second round was launched on March 13, 2025, and completed on March 20, 2025.

**Table 3:** Statements proposed to the panel of clinical experts at the second round of the Delphi process

S11	Pediatric endocrinologists are skilled in managing children with short stature of both genetic and non-genetic origin, offering the best care through a personalized diagnostic and therapeutic approach. Pediatric Endocrinology centers provide all the needed professionals, including clinical and laboratory geneticists, for the best diagnostic and therapeutic management. **Any pediatrician should refer a child with short stature and suspected genetic causes to a Pediatric Endocrinology center, rather than to laboratory or clinical geneticist.**
S12	Short stature may be the only clinical feature in girls with Turner syndrome, a condition characterized by the presence of only one X chromosome and the complete or partial absence of the second sex chromosome. Unexplained short stature, left-sided outflow congenital heart defects, unexplained delay in puberty, primary and secondary amenorrhea, failure to progress puberty and characteristic facial and physical features are all indications for genetic testing. Other minor and less specific features may be present including sensorineural hearing loss, renal abnormalities, Madelung deformity, neurocognitive problems, and additional congenital heart defects. **Any pediatrician may either order a karyotype (with a minimum of 30 metaphases counted) in girls with unexplained short stature**,** especially if associated with other characteristic signs of Turner syndrome before referring her to Pediatric Endocrinology center or refer the girl straight to a Pediatric Endocrinology center.**
S14	Due to the progressive reduction in costs and the increasing availability of easily accessible laboratory facilities, genetic testing is becoming central to the evaluation of a child with short stature. WES significantly improves the diagnostic yield and, as a result, enhances care for patients with short stature. A wide range of genetic variants have been linked to hypopituitarism. The most common pathogenic genetic variants causing non-growth hormone (GH) dependent short stature include the ACAN, BMP2, FBN1, FGFR3, IHH, IGF-I receptor, NPPC, NPR2, PAPPA2, PTPN11, and SHOX genes. **Every pediatrician should recognize the importance of genetic testing when assessing a child with short stature and stay informed on the most common genetic tests available in the context of any continuous education program.**

In the first round, as for the S11 statement, 77.1% expressed strong agreement, 17.1% moderate agreement, with a minority of respondents (5.7%) indicating no agreement. As for the S12 statement, most of respondents (77.1%) expressed strong agreement, 20.0% moderate agreement, with a minority of respondents (2.9%) indicating no agreement. Finally, as for the S14 statement, most of respondents (77.1%) expressed strong agreement and the rest (22.9%) moderate agreement.

**Fig. 2:**
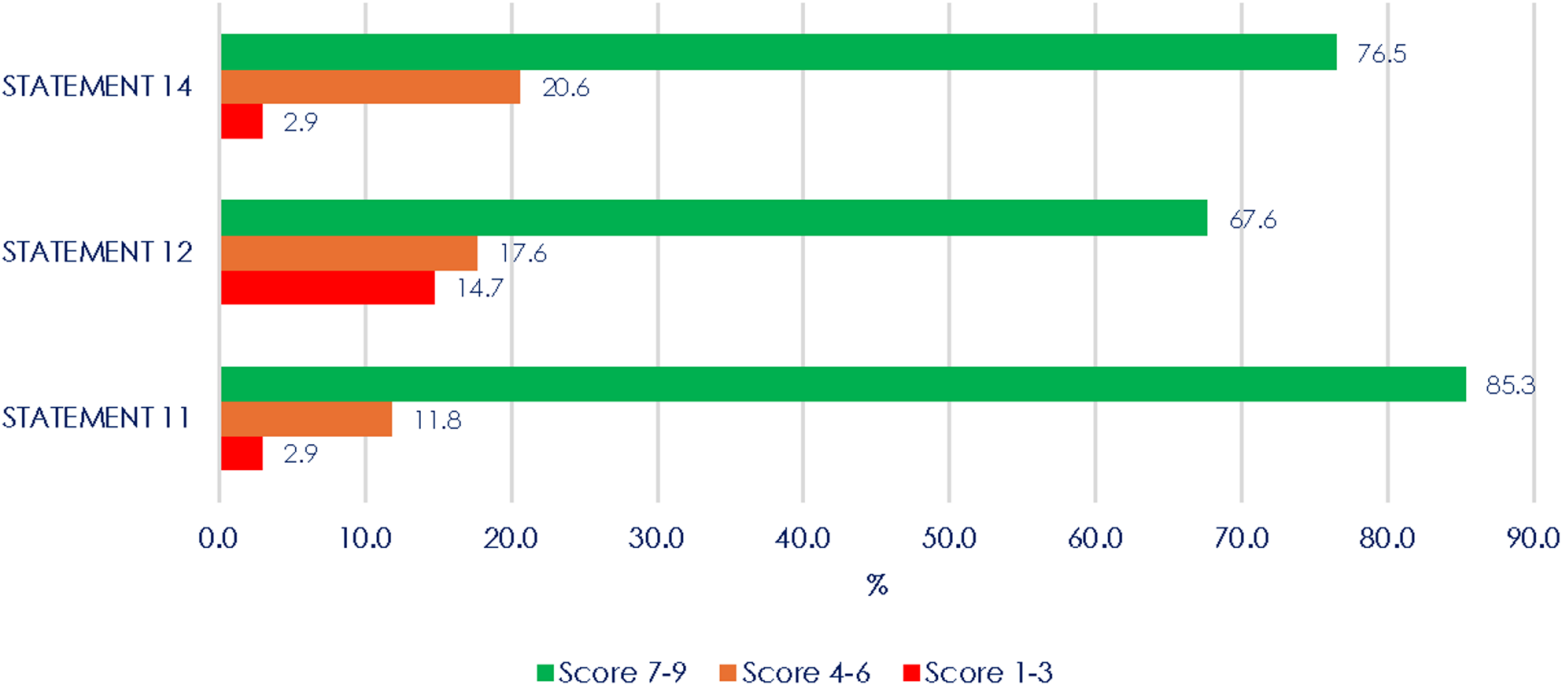
Scores of the proposed statements (second Delphi round). Results are presented in %

All the 34 clinical experts who had answered in the first round completed the second-round survey (response rate: 100%). Strong agreement was recorded for one of the three re-proposed statements (Fig. 2). In conclusion, consensus was not reached only for two statements.

## Discussion

This Delphi panel study reached a consensus among pediatricians and pediatric endocrinologists on relevant aspects of the management of short stature, particularly focusing on clinical, biochemical, imaging, and genetic domains. Nevertheless, this Delphi process also revealed uncertain issues, particularly related to the role of genetic testing in the diagnostic workup of patients with short stature.

### Topic 1. Clinical evaluation (statements 1–4)

All the 4 statements on the clinical evaluation of children with short stature reached a consensus. When considered in aggregate, these statements highlight the importance of early recognition of growth abnormalities to avoid late diagnosis. Scientific panel members agreed that primary care settings provide a unique opportunity to promote health care.

Early identification of abnormal growth patterns is crucial for the timely diagnosis of short stature in pediatric patients. This process relies on objective anthropometric thresholds, the mid-parental target range and the reduction in height velocity [12, 13]. Therefore, in statement 1 the scientific panel proposed to include all the 3 auxological parameters (height, height velocity, target height) to improve the early detection of children with stunted growth and potential abnormal short stature. Head circumference, particularly if below the 3rd percentile or less than − 2 SDS for age, may provide additional support for diagnosing of growth disorders or neurodevelopmental conditions, primarily when associated with other clinical signs. A head circumference z-score below − 2 should be used as a diagnostic threshold. However, whether absolute or relative, macrocephaly may also be present in several skeletal dysplasia or syndromic conditions associated with proportionate or disproportionate short stature [14, 15]. For preterm infants, growth should be evaluated using corrected age until at least 24 months post-term, and the use of specific growth charts (e.g., Fenton or INTERGROWTH-21st) is recommended in the first year [16, 17].

Besides the statement, the scientific board suggested how to monitor child growth during pediatric age. In infancy (0–12 months), the length should be measured at routine pediatric visits, typically at 2, 4, 6, 9, and 12 months of age and height velocity should be calculated. In toddlers (12–24 months), the length should also be monitored regularly—commonly at 15, 18, and 24 months of age—along with any additional assessments prompted by clinical concerns. From 2 years of age onward, standing height should be measured at least once every 12 months. In specific situations, such as when height velocity is below − 1 SDS or during the peri-pubertal period, growth monitoring should be performed every 6 months [18]. The frequency and timing of assessments should take into account the child’s sex, given the different tempo and pattern of pubertal development in males and females.

As with infants born appropriate for gestational age, SGA babies should undergo growth assessments during routine pediatric visits. Similarly, preterm babies with intra-uterine and/or extra-uterine growth retardation, require regular and serial growth monitoring and tailored evaluation strategies to ensure the timely identification of persistent growth impairment and to guide appropriate clinical interventions [19].

### Topic 2. Biochemical assessment (statements 5–6)

Both statements on the biochemical evaluation of children with short stature reached a consensus. Laboratory tests should be used to assess the major causes of short stature in children. Previous consensus documents on the topic of short stature recommend routine laboratory screening for occult disease in asymptomatic short children [2, 3, 20]. Laboratory screening to detect a subclinical chronic illness that initially can present as isolated growth failure should be performed in primary care in order to not miss clinically relevant diagnosis. Many non-endocrine conditions should be investigated and ruled out by the primary care physician, before sending patients to the pediatric endocrinology center.

### Topic 3. Imaging domain (statements 7–10)

All the 4 statements about the imaging evaluation of children with short stature reached consensus. The scientific panel members confirmed the remarkable importance of radiographic bone age assessment as a valuable tool that aids clinicians in making informed decisions about potential treatments and in predicting a child’s future growth potential. Authors also suggest pediatric endocrinologists should review radiographic images directly, not just the reports, given the variability of radiologist reports for bone age assessment. An accurate bone age assessment by experienced pediatric endocrinologists is essential.

While a skeletal survey is not part of the routine initial evaluation for short stature, it is suggested as an important diagnostic tool when skeletal dysplasia is suspected, particularly in children with disproportionate short stature or other physical signs suggestive of bone abnormalities [21]. Authors also suggested that x- ray images should always be available as mild skeletal deformities might not be recognized by automatic software, artificial intelligence-based methods on not enough experienced radiologists. In cases with shortening or alterations in the appendicular skeleton, the central body regions to be radiographed include the long bones, knees, hands, wrist, and feet. For alterations in the axial skeleton, key body regions to be radiographed are the skull, chest, cervical and thoracolumbar spine, and pelvis [22]. It is important to note that a skeletal survey is a radiologically intensive procedure, typically used when other diagnostic methods, such as genetic testing or bone age assessment, are inconclusive, or when specific skeletal abnormalities are suspected based on clinical presentation.

The scientific board recommended MRI without contrast using T2-DRIVE sequences for the evaluation of suspected hypopituitarism in pediatric patients, minimizing unnecessary contrast exposure. This approach is an essential part of the workup in children, neonates, and adolescents with suspected hormonal deficiencies or growth disturbances [23]. Comprehensive brain imaging is valuable because hypothalamic-pituitary disorders may sometimes be associated with other brain abnormalities. For example, genetic conditions like septo-optic dysplasia or other structural brain malformations may affect both the hypothalamus and other regions of the brain. A comprehensive brain scan ensures that any additional anomalies are identified and addressed.

Finally, the scientific panel members highlighted the importance of prenatal diagnosis of both skeletal dyplasia and septo-optic dysplasia. These conditions can be suspected in utero through routine ultrasound. If the routine obstetric ultrasound detects short limbs, suggesting fetal bone dysplasia, a further assessment with more detailed two- dimensional ultrasound (2D- US), three- dimensional US (3D- US), MRI, and computed tomography is recommended [24]. Agenesis of the septum pellucidum without significant brain malformations should raise strong suspicion for septo-optic dysplasia. In such cases, evaluation of the optic tracts, optic nerves, and optic chiasm using two-dimensional/three-dimensional (2D/3D) ultrasound or fetal MRI is recommended [25].

### Topic 4. Genetic investigation (statements 11–14)

Only one statement (Statement 13) on this domain reached the consensus during the first Delphi round. This statement recognized the role of genetic testing when a detailed history, physical examination, laboratory results, and radiological findings point out to a possible genetic cause of short stature. A growing number of genetic causes of short stature affecting either the growth plate or the pituitary-IGF axis are now recognized and approximately half of children previously diagnosed with familial or idiopathic short stature, if properly screened, may receive a molecular diagnosis [26].

Statement 11, involving the role of Pediatric Endocrinology centers in the management of children with short stature, reached the consensus in the second Delphi round. The scientific panel suggested that tertiary Pediatric Endocrinology centers provide all the necessary competences for this stepwise diagnostic approach ultimately leading to genetic tests in selected cases. Genetic and/or epigenetic tests should be performed in the diagnostic assessment of specific groups of children whose phenotype suggests a high likelihood of a genetic cause. For this reason, an experienced Pediatric Endocrinologist should be the case manager of children with severe short stature potentially secondary to a genetic defect [3]. Genetic characterization of severe forms of short stature may allow to: (1) start growth hormone treatment in specific disorders; (2) provide information about potential associated conditions that may require treatment; (3) prevent growth hormone treatment in cancer predisposing genetic alterations (e.g. Bloom syndrome); (4) provide a definitive diagnosis thus satisfying patients, caregivers and physicians; (5) lead to the identification of additional affected family members; (6) give genetic counselling and, finally, (7) open avenues for new treatment modalities based on genomics (e.g. C-natriuretic peptide analogue for achondroplasia).

Two statements (12 and 14) regarding the prescription of karyotype by any pediatrician and the importance of genetic testing when assessing a child with short stature did not reach consensus. The scientific board agreed that the median age at diagnosis of Turner syndrome is still high and improved awareness of the syndrome from pediatricians and family physicians will result in early diagnosis thus enabling timely intervention and improving clinical outcomes [27]. In the preliminary diagnostic work-up to be performed before referring the patient to a Pediatric Endocrinology center, pediatricians may request karyotype analysis along with biochemical and endocrine tests in girls with short stature and other clinical features suggestive for Turner syndrome. However, 37.4% of Delphi members did not agree. We can speculate that some Delphi panel members who are not primary involved in Pediatric Endocrinology Centers are less confident in prescribing genetic tests. Moreover, regional and national prescribing rules and reimbursement issues are currently an obstacle for many pediatricians. Similarly, 24.5% of Delphi members did not agree with Statement 14, which pointed out that every pediatrician should recognize the importance of genetic testing when assessing a child with short stature and stay informed on the most common genetic tests available in the context of any continuous education program. Genomic technology continues to advance and is being applied in multiple clinical settings, yielding molecular insights into several disorders including short stature. A growing number of genetic causes of short stature affecting either the growth plate or the pituitary-IGF axis are now recognized and approximately half of children previously diagnosed with familial or idiopathic short stature, if properly screened, may receive a molecular diagnosis [26, 28].

This genetic revolution is a challenge for primary care clinicians who will face new diagnostic and therapeutic approaches to care and will need to critically incorporate in their daily practice the results stemmed from increasingly sophisticated genomic tools. Therefore, the scientific panel recognized the importance of genetic education and it suggested that it must be incorporated into undergraduate, graduate, and continuing medical education [29].

### Strengths and limitations

The strengths of this Consensus Statement include the expert selection based on expertise through the inclusion of pediatric endocrinologists, family pediatricians and patient representatives to ensure the patient view was taken into consideration in this consensus. The Delphi panel was also composed by multidisciplinary experts (Pediatricians, Pediatric Endocrinologists, Family Pediatricians), from all the Italian Regions. The widely accepted Delphi approach was used across multiple rounds with a predefined threshold, to reach a consensus on the given statements. Moreover, a systematic literature review was conducted to guide the scientific board. Owing to the practical nature of the statements, limited published evidence was available to support individual recommendations.

## Conclusions

This consensus provides valuable insights and recommendations to guide pediatricians in approaching a child with short stature. By addressing clinical, biochemical, imaging and genetic domains this consensus aims to enhance short stature management. The genetic approach for evaluating short stature and the role of genetic testing remain challenging, however, recent developments in this field need to be specifically addressed in the near future.

## Appendix 1

Panel of participating experts (*n* = 34)

**Table:** 

DELPHI PANEL MEMBERS	SPECIALITY	AFFILIATION
AGOSTINIANI RINO	Pediatrician	Department of Pediatrics, ASL Toscana Centro, Florence, Italy
AVERSA TOMMASO	Pediatric Endocrinologist	Department of Human Pathology of Adulthood and Childhood, University of Messina, Messina, Italy. Pediatric Unit, “G. Martino” University Hospital, Messina, Italy.
BARONIO FEDERICO	Pediatric endocrinologist	PEDIATRIC UNIT, IRCCS Azienda Ospedaliero-Universitaria di Bologna, Italy
BELLONE SIMONETTA	Pediatric endocrinologist	Division of Pediatrics, Dept of Health Sciences, University of Piemonte Orientale, Novara, Italy
BIZZARRI CARLA	Pediatric Endocrinologist	Endocrinology Unit “Bambino Gesù” Children’s Hospital- IRCSS, Rome, Italy
BONA GIANNI	Pediatric Endocrinologist	Pediatric Endocrinologist, Honorary Professor, Universilty of Piemonte Orientale, Novara, Italy.
BOZZOLA ELENA	Pediatrician	Pediatric Unit, IRCCS Bambino Gesù Children Hospital, Rome, Italy
CAPALBO DONATELLA	Pediatric Endocrinologist	Unit of Pediatric Endocrinology Department of Medical Translational Sciences, University Federico II of Naples , Italy
CAPPA MARCO		
CAVARZERE PAOLO	Pediatric Endocrinologist	Department of Mother and Child, Pediatric Unit B, University Hospital of Verona, Verona, Italy
CHIARITO MARIANGELA	Pediatric Endocrinologist	Pediatric Hospital Giovanni XXIII Department of Precision and Regenerative Medicine and Ionian Area, University of Bari “Aldo Moro
CORICA DOMENICO	Pediatric Endocrinologist	Department of Human Pathology of Adulthood and Childhood, University of Messina, Messina, Italy. Pediatric Unit, “G. Martino” University Hospital, Messina, Italy.
DELVECCHIO MAURIZIO	Pediatric Endocrinologist	Department of Biotechnological and Applied Clinical Sciences, University of L’Aquila, L’AQUILA
DEODATI ANNALISA	Pediatric Endocrinologist	Department of Systems Medicine, University of Rome "Tor Vergata” Endocrinology & Diabetes Unit “Bambino Gesù” Children’s Hospital- IRCSS, Italy
DI MASE RAFFAELLA	Pediatric Endocrinologist	Maternal and Child Health Department – Federico II University Hospital of Naples, Italy
FRANCESCHI ROBERTO	Pediatric Endocrinologist	Department of Pediatrics, Santa Chiara Hospital of Trento, Azienda Provinciale per i Servizi Sanitari della Provincia Autonoma di Trento, Trento, Italy Centre for Medical Sciences, University of Trento, Trento, Italy
GERDI TULI	Pediatric Endocrinologist	Pediatric Endocrinology Unity, Regina Margherita Hospital, Turin. Department of Pediatric Science, University of Turin, Italy
GIACOMOZZI CLAUDIO	Pediatric Endocrinologist	Unit of Pediatrics, Department of Maternal and Child Health, Asola Hospital, ASST-Mantova, Italy
GRANDONE ANNA	Pediatric Endocrinologist	Department of Woman, Child, general and Specialized Surgery; University of Campania “L. VANVITELLI”, Italy
GRECO LUIGI	Family Pediatrician	ASST Papa Giovanni XXIII, Bergamo, Italy
GUAZZAROTTI LAURA	Pediatric Endocrinologist	Pediatric Endocrinology Unit, University Hospital of Padova, Padova, Italy.
IBBA ANASTASIA	Pediatric Endocrinologist	Pediatric Endocrinology and Neonatal Screening Unit, Microcitemico “A.Cao” Children’s Hospital, Cagliari, Italy
IUGHETTI LORENZO	Pediatric Endocrinologist	Pediatric Unit, Department of Medical and Surgical Sciences of Mother, Children and Adults, University of Modena and Reggio Emilia, Italy
LOCHE SANDRO	Pediatric Endocrinologist	Research Area for Innovative Therapies in Endocrinology Bambino Gesù Children Hospital, IRCCS, Italy
MADEO SIMONA	Pediatric Endocrinologist	Auxology and pediatric rare diseases, pediatric endocrinology. Department of Medical and Surgical Sciences for Mother, Children and Adults, Pediatric Unit, University of Modena and Reggio Emilia, Modena, Italy
MASTROMAURO CONCETTA	Pediatric Endocrinologist	Department of Pediatrics, University of Chieti, Chieti, Italy.
PATTI GIUSEPPA	Pediatric Endocrinologist	Department of Pediatrics, IRCCS Istituto Giannina Gaslini, University of Genova, Genova, Italy; Department of Neuroscience, Rehabilitation, Ophthalmology, Genetics, Maternal and Child Health, University of Genova, Genova, Italy.
PICCA MARINA	Family Pediatrician	ASST Fatebenefratelli Sacco, Milan, Italy
POZZOBON GABRIELLA	Pediatric Endocrinologist	Pediatric Department, San Raffaele Hospital, 20,132 Milan, Italy.
SCARPATO ELENA	Pediatrician	Department of Translational Medical Sciences - Section of Pediatrics, University of Naples Federico II, Naples - Italy
SECCO ANDREA	Pediatric Endocrinologist	Pediatric and Pediatric Emergency Unit, Children Hospital, Azienda Ospedaliera Universitaria SS Antonio e Biagio e C. Arrigo, Alessandria, Italy.
SOLLAI SARA	Pediatrician	Department of Pediatrics, Santa Maria Annunziata Hospital, Florence, Italy
TORNESE GIANLUCA	Pediatric Endocrinologist	Department of Medical Sciences, University of Trieste, Trieste, Italy Institute for Maternal and Child Health IRCCS “Burlo Garofolo”, Trieste, Italy
VANNELLI SILVIA	Pediatric Endocrinologist	Centre of Auxology- Department of Pediatric Endocrinology Regina Margherita Children Hospital, Turin, Italy

## Supplementary Information

Below is the link to the electronic supplementary material.

Supplementary Material 1

## Data Availability

Materials about this study can be obtained from the corresponding author on reasonable request.

## Abbreviations

ASP: Agenesis of the septum pellucidum
BA: Bone age
CDC: Centers for Disease Control and Prevention
GH: Growth hormone
GHD: Growth hormone deficiency
GV: Growth velocity
ISPED: Italian Society for Pediatric Endocrinology and Diabetology
MRI: Magnetic resonance imaging
S(s): Statement(s)
SGA: Small for gestational age
SDs: Skeletal dysplasias
SDS: Standard deviation score
SOD: Septo-optic dysplasia
WES: Whole-exome sequencing
WHO: World Health Organization
2D- US: Two- dimensional ultrasound
3D- US: Three- dimensional ultrasound
