# Supplementary material for: Towards a shared diagnostic approach for pediatric short stature: a Delphi consensus of the Italian Society of Pediatrics and the Italian Society for Pediatric Endocrinology and Diabetology
Source: Ital J Pediatr. 2026 Jan 10;52:26. doi: 10.1186/s13052-025-02190-6 (PMC12882444; doi:10.1186/s13052-025-02190-6)
Supplement: Supplementary file 1 — Supplementary Material 1 [file 13052_2025_2190_MOESM1_ESM.docx]

| *Supplementary File 1 - Search strategy* | | |
| --- | --- | --- |
| PubMed, 30.03.2024 | | |
| Search | Query | Results |
| #1 | guideline[Title] OR guidelines[Title] OR consensus[Title] OR recommendation[Title] OR recommendations[Title] OR "guideline"[Publication Type] OR "practice guideline"[Publication Type] OR "consensus development conference"[Publication Type] | 195,898 |
| #2 | short stature[Title/Abstract] | 14,051 |
| #3 | #1 AND #2 | 84 |
| #4 | #1 AND #2 Filters: English | 74 |
| Embase, 30.03.2024 | | |
| Search | Query | Results |
| #1 | guideline:ti OR guidelines:ti OR consensus:ti OR recommendation:ti OR recommendations:ti | 226,561 |
| #2 | short stature:ti,ab | 22,822 |
| #3 | #1 AND #2 | 91 |
| #4 | #1 AND #2 AND [english]/lim | 77 |
| Web of Science, 30.03.2024 | | |
| Search | Query | Results |
| #1 | TI= (guideline OR guidelines OR consensus) | 167,345 |
| #2 | TS= (short stature) | 16,344 |
| #3 | #1 AND #2 | 53 |
| #4 | (#1 AND #2) AND English (Languages) | 51 |
| Google Scholar, 02.04.2024 | | |
| Search | Query | Results |
| #1 | (guideline OR guidelines OR consensus) AND (short stature) | First 50 records examined |

| *Supplementary File 2- PRISMA 2020 Flow Diagram* |
| --- |
| **Identification of studies via databases and search engines**  **Identification**  Records removed *before screening*:  Duplicate records removed (n= 111)  Records identified from:  Databases (n = 202)   - PubMed (n = 74) - Embase (n = 77) - Web of Science (n = 51)   Search engines (n = 50)   - Google Scholar (n = 50)   Records screened  (n = 141)  Records excluded**  (n = 121)  Reports sought for retrieval  (n = 20)  Reports not retrieved  (n = 2)  **Screening**  Reports assessed for eligibility  (n =18)  Reports excluded:12  Study Design (n =12)  Studies included in review  (n =6)  Reports of included studies  (n =6)  **Included** |

| *Supplementary File 3*– Excluded studies | |
| --- | --- |
| **Study** | **Reason for exclusion** |
| (1996). Growth Standards, Bone Maturation and Idiopathic Short Stature. Proceedings of the 1st KIGS Expert Meeting. Positano, Italy, November 10-11, 1995. Horm Res 45 Suppl 2: 1-67. | Full-text not retrieved |
| (2009). Erratum: ACMG practice guideline: Genetic evaluation of short stature (Genetics in Medicine (2009) 11 (465-470)). Genetics in Medicine 11(10): 765. | Study design: erratum (Seaver 2009) |
| Al Herbish, A. S., et al. (2016). Diagnosis and management of growth disorders in Gulf Cooperation Council (GCC) countries: Current procedures and key recommendations for best practice. Int J Pediatr Adolesc Med 3(3): 91-102. | Study design: Review |
| Boguszewski, M. C., et al. (2011). Latin American consensus: children born small for gestational age. BMC Pediatr 11: 66. | Study design: consensus SGA |
| Chernausek, S. D. (2002). Development of a consensus statement on management of short children born small for gestational age. Journal of Pediatric Endocrinology & Metabolism 15: 1275-1275. | Abstract not retrieved |
| Evans, C. and J. W. Gregory (2004). The investigation of short stature: a survey of practice in Wales and suggested practical guidelines. J Clin Pathol 57(2): 126-130. | Study design: survey |
| Grote, F. K., et al. (2008). The diagnostic work up of growth failure in secondary health care; an evaluation of consensus guidelines. BMC Pediatr 8: 21. | Study design: observational retrospective study |
| Grote, F. K., et al. (2008). Developing evidence-based guidelines for referral for short stature. Arch Dis Child 93(3): 212-217. | Study design: study proposing screening rules based on auxological criteria for 4 patient groups and 3 control groups. |
| Hokken-Koelega, A. C. S., et al. (2023). International Consensus Guideline on Small for Gestational Age: Etiology and Management From Infancy to Early Adulthood. Endocr Rev 44(3): 539-565. | Study design: consensus SGA |
| Lee, P. A., et al. (2003). International Small for Gestational Age Advisory Board consensus development conference statement: management of short children born small for gestational age, April 24-October 1, 2001. Pediatrics 111(6 Pt 1): 1253-1261. | Study design: consensus SGA |
| Melver, C. W. (2009). Reply to "Practice guidelines for short stature". Genet Med 11(10): 766; author reply 766. | Study design: reply (Seaver 2009) |
| Mulligan, J., et al. (1998). Growth monitoring: Testing the new guidelines. Arch Dis Child 79(4): 318-322. | Study design: study assessing the impact of recent UK Joint Working Group on Child Health Surveillance guidelines recommending that all children be measured at age 5 and again between ages 7 and 9 to determine how many children of normal school age could be referred for specialist assessment. |
| Seaver, L. F., et al. (2009). ACMG practice guideline: Genetic evaluation of short stature (vol 11, pg 465, 2009). Genetics in Medicine 11(10): 765-765. | Study design: abstract (Seaver 2009) |
| Stalman, S. E., et al. (2015). Application of the Dutch, Finnish and British Screening Guidelines in a Cohort of Children with Growth Failure. Horm Res Paediatr 84(6): 376-382. | Study design: study evaluating the application of 3 guidelines: Grote 2008, Saari 2012 (Turner syndrome) and Hall 2000 (consensus on growth monitoring) |
